# Genotoxic Effect of *N*-Hydroxy-4-Acetylaminobiphenyl on Human DNA: Implications in Bladder Cancer

**DOI:** 10.1371/journal.pone.0053205

**Published:** 2013-01-31

**Authors:** Uzma Shahab, Moinuddin, Saheem Ahmad, Kiran Dixit, Safia Habib, Khursheed Alam, Asif Ali

**Affiliations:** Department of Biochemistry, J. N. Medical College, Faculty of Medicine, Aligarh Muslim University, Aligarh, India; Aligarh Muslim University, India

## Abstract

**Background:**

The interaction of environmental chemicals and their metabolites with biological macromolecules can result in cytotoxic and genotoxic effects. 4-Aminobiphenyl (4-ABP) and several other related arylamines have been shown to be causally involved in the induction of human urinary bladder cancers. The genotoxic and the carcinogenic effects of 4-ABP are exhibited only when it is metabolically converted to a reactive electrophile, the aryl nitrenium ions, which subsequently binds to DNA and induce lesions. Although several studies have reported the formation of 4-ABP-DNA adducts, no extensive work has been done to investigate the immunogenicity of 4-ABP-modified DNA and its possible involvement in the generation of antibodies in bladder cancer patients.

**Methodology/Principal Findings:**

Human DNA was modified by N-hydroxy-4-acetylaminobiphenyl (*N*-OH-AABP), a reactive metabolite of 4-ABP. Structural perturbations in the *N*-OH-AABP modified DNA were assessed by ultraviolet, fluorescence, and circular dichroic spectroscopy as well as by agarose gel electrophoresis. Genotoxicity of *N*-OH-AABP modified DNA was ascertained by comet assay. High performance liquid chromatography (HPLC) analysis of native and modified DNA samples confirmed the formation of *N*-(deoxyguanosine-8-yl)-4-aminobiphenyl (dG-C8-4ABP) in the *N*-OH-AABP damaged DNA. The experimentally induced antibodies against *N*-OH-AABP-modified DNA exhibited much better recognition of the DNA isolated from bladder cancer patients as compared to the DNA obtained from healthy individuals in competitive binding ELISA.

**Conclusions/Significance:**

This work shows epitope sharing between the DNA isolated from bladder cancer patients and the *N*-OH-AABP-modified DNA implicating the role of 4-ABP metabolites in the DNA damage and neo-antigenic epitope generation that could lead to the induction of antibodies in bladder cancer patients.

## Introduction

**Figure 1:**
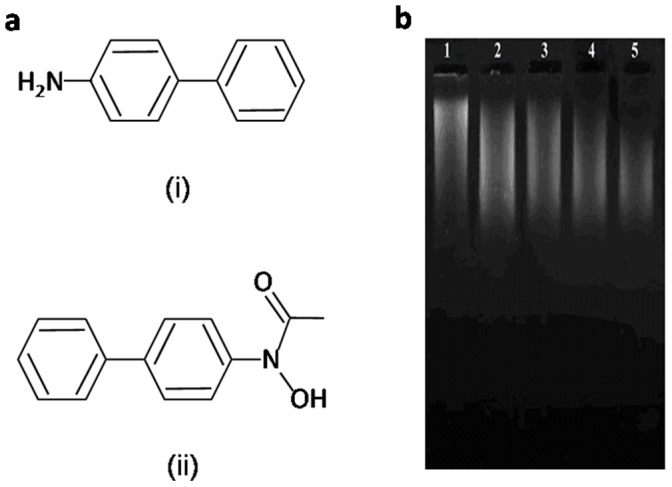
Chemical structure of 4-ABP and *N*-OH-AABP [a]. Agarose gel electrophoresis of native and *N*-OH-AABP modified human DNA **[b]**. DNA samples from lanes 2–5 were treated with increasing concentration of *N*-OH-AABP. Lane 1: Native human DNA; Lane 2: Native human DNA with 0.378 mM *N*-OH-AABP; Lane 3: Native human DNA with 0.757 mM *N*-OH-AABP; Lane 4: Native human DNA with 1.136 mM *N*-OH-AABP; Lane 5: Native human DNA with 1.515 mM *N*-OH-AABP.

The DNA of cells exposed to a chemical carcinogen nearly always contains a set of structurally diverse carcinogen-nucleotide adducts [1]. It is suspected that misreplication or misrepair of a subset of these adducts gives rise to mutations, which in turn may be the genetic precursors of the cancer phenotype. One such carcinogen is 4-aminobiphenyl, an aromatic amine. These are capable of inducing a variety of toxic effects, including cancer and met-hemoglobinemia [2]. 4-ABP is an environmental and occupational contaminant generated mainly from cigarette smoke, combustion of fossil fuels and from rubber, coal, textile and print industries [3]–[5]. 4-ABP has been shown to be a major etiological agent of human bladder cancer, and also a potent urinary bladder carcinogen in experimental animals [6], [7]. 4-ABP residues have been reported in haemoglobin and in the DNA isolated from the bladder of smokers [8], [9]; potentially implicating 4-ABP in the etiology of bladder cancer. The chemical structure of 4-ABP and its physiological metabolite, *N*-OH-AABP, is placed as figure 1A.

The genotoxic and carcinogenic effects are exhibited when 4-ABP is metabolically converted to a reactive electrophile. The primary step is *N*-oxidation of arylamines and arylacetamides by specific cytochrome *P*450 (CYP1A2) in hepatic tissues [10], followed by conjugation of the *N*-hydroxyl function with acetate, sulfate, or glucuronate [11]–[14]. The activated electrophilic derivatives of 4-ABP exert their genotoxic effect through interaction with DNA forming adducts that have been reported for a role in bladder carcinogenesis both in experimental animals [15]–[17] and humans [18], [19]. DNA adducts have been also reported upon exposure of human bladder cells to *N*-OH-ABP, *N*-OH-AABP and *N*-OAc-AABP [20]–[22]. However, the major (80%) DNA adduct formed has been identified as *N*-(deoxyguanosin-8-yl)-4-aminobiphenyl (dG-C8-ABP) [19]. Even though the major DNA lesions are a result of covalent adduct formation, these metabolites also cause oxidative DNA damage producing reactive oxygen species [23] that ultimately generate DNA strand breaks and base modifications such as 8-oxo-guanine and related products [24], [25].

The mode of action of various carcinogens is better ascertained by analysing their genotoxicity [26]. The present study reports structural changes in human DNA upon incubation with *N*-OH-AABP for 24 h. The modified DNA was characterised by various spectroscopic techniques, gel electrophoresis and thermal denaturation studies. The genotoxicity of *N*-OH-AABP was ascertained through comet assay. Antibodies against *N*-OH-AABP-DNA were induced in female rabbits and used as an immunochemical probe to detect the lesions, caused by 4-ABP metabolites, in the DNA of bladder cancer patients.

*Ethics Statement-* This study was approved by Institutional Animal Ethics Committee of J.N. Medical College, AMU, Aligarh, India (permit no. 401/CPCSEA). Blood samples from patients and healthy individuals were collected after informed verbal consent.

## Materials and Methods

*N*-OH-AABP was from Midwest research institute Kansas. Human placental DNA, methylated bovine serum albumin (MBSA), ethidium bromide, Protein A-Agarose (2.5 ml pre-packed column), anti-rabbit IgG alkaline phosphatase conjugates, p-nitrophenyl phosphate, Tween-20, Freund’s complete & incomplete adjuvants, histopaque 1077, low melting point agarose (LMPA), RPMI 1640, Triton-X 100, trypan blue, and phosphate buffered saline (PBS) Ca^2+^ and Mg^2+^ were from Sigma Chemical Company, USA. Polystyrene microtitre flat bottom ELISA plates were from Nunc (Denmark). All other chemicals and reagents used were of highest analytical grade.

### Modification of Human Placental DNA

Human placental DNA (15 µM) was modified by incubating at 37°C for 24 h with varying concentrations (0.378 mM, 0.757 mM, 1.136 mM and 1.515 mM) of *N*-OH-AABP in DMSO. Unbound constituents were removed by extensive dialysis against sodium phosphate buffer (10 mM, pH 7.4) containing 150 mM NaCl.

### Agarose Gel Electrophoresis

The change in electrophoretic pattern of native and modified DNA was observed on 1% agarose at 30 mA for 2 h in TAE buffer (40 mM Tris–acetate, 2 mM EDTA, pH 8.0). The gels were stained with ethidium bromide and visualized under UV light.

### Isolation of Lymphocytes

Heparinized blood samples (2 ml) from healthy donors and patients were diluted suitably in Ca^2+^ and Mg^2+^ free PBS. Lymphocytes were isolated from blood using Histopaque 1077 and the cells were finally suspended in RPMI 1640.

### Viability Assessment of Lymphocytes

The lymphocytes were checked for their viability before the start and after the end of the reaction using Trypan Blue Exclusion test [27]. The viability of the cells was found to be greater than 93%.

### Lymphocyte Treatment

Lymphocytes (1×10^5^ cells) were exposed to different concentrations of N-OH-AABP (0.378 mM, 0.757 mM, 1.136 mM, 1.515 mM) in a total reaction volume of 1 ml and incubated at 37°C for 90 min. After the incubation, the mixture was centrifuged at 4000 rpm, supernatant discarded and the pelleted lymphocytes were resuspended in 100 µl of PBS (Ca^2+^ and Mg^2+^ free) and processed further for Comet assay.

### Comet Assay

Comet assay was performed as per the protocol given in an earlier publication from our lab [28].

### Physicochemical Analysis

The fluorescence analysis was carried out on Shimadzu (RF-5301-PC) spectrofluorophotometer. The samples were excited at 325 nm and emission profile was recorded in the 500 to 700 nm range. Circular dichroism (CD) measurements of native and modified human DNA were recorded on Jasco J-815 spectropolarimeter in the 220–400 nm wavelength range. All the scans were recorded at an interval of 1 nm.

### High Performance Liquid Chromatography

High performance liquid chromatography (HPLC) analysis of native and modified DNA was performed on Biologic Duo flow system, BioRad, (USA) equipped with UV-Visible multi wavelength detector. The absorbance was monitored at 245 nm. Briefly 50 µM DNA sample was hydrolyzed in 0.1 N HCl (1 ml/mg DNA) at 100°C for 30 min (in order to release the bases) and dried under vacuum. The bases were dissolved in 50 µl of 0.1 M Tris HCl buffer, pH 8.5 and filtered through 0.42 µM Milex, disposable syring filter before loading on C-18 reversed phase column (Supelco, Discovery 25 cm × 4.6 mm). The eluent buffer was 12.5 mM citric acid, 25 mM sodium acetate, 25 µM ethylenediaminetetraacetate (EDTA), pH 5.2 and a flow rate of 1 ml/min was maintained throughout.

### Immunization Schedule

Random bred, New Zealand White (NZW) female rabbits were immunized as described previously [28], [29]. Briefly, rabbits (n  = 4; two each for native and modified DNA) were immunized intramuscularly at multiple sites with 50 µg of respective antigens complexed with methylated BSA in 1∶1 ratio (w/w) and emulsified with an equal volume of Freund’s adjuvant.

### Purification of Antibodies

Immunoglobulin G (IgG) was affinity purified from preimmune and immune sera on a protein A-Agarose column [30]. The homogeneity of isolated IgG was ascertained by 7.5% SDS–PAGE.

### DNA Isolation from Human Blood

Blood samples were collected in EDTA vials from different bladder cancer patients and normal healthy individuals. Lymphocyte DNA was isolated as per the manufacturer’s instructions. 5 ml of blood was added to 500 µl of Qiagen protease and then buffer AL (6 ml) was mixed followed by vigorous shaking. The mixture was incubated at 70°C for 10 min and 5 ml of ethanol (98%) was added to the sample and mixed by inverting the tube and vigorous shaking. The solution was carefully transferred onto QIAamp Maxi column placed in a 50 ml centrifuge tube and centrifuged at 1850×g (3000 rpm) for 3 min. The filtrate was discarded and 5 ml of Buffer AW2 was added and again centrifuged at 4500×g (5000 rpm) for 1 min. Again 5 ml of AW2 buffer was added and centrifuged at 4500×g (5000 rpm) for 15 min and the filtrate was discarded. Then 600 µl of buffer AE was poured directly onto the membrane of the column which was later incubated for 5 min, and centrifuged at 4500×g (5000 rpm) for 2 min. The eluent was reloaded onto the membrane of the column, incubated for 5 min, and centrifuged at 4500×g (5000 rpm) for 5 min. The eluent containing DNA was air dried and dissolved in PBS, pH 7.4. The purity and concentration of DNA preparation was ascertained by A_260_ and A_280_ measurements.

### ELISA

Specific binding of antibodies was ascertained in competitive binding assay [31]. Percent inhibition was calculated from the given formula:

Percent inhibition  = 1− (A_inhibited_/A_uninhibited_) ×100.

## Results and Discussion

The gel pattern (Figure 1B) shows an increase in the mobility of modified DNA with increasing concentration of *N*-OH-AABP (lanes 2–5). The maximum mobility was observed with 1.515 mM *N*-OH-AABP; and any further increase in its concentration resulted in complete loss of DNA structure. The increase in mobility of the N-OH-AABP treated DNA may be due to the generation of single strand breaks which may result in the formation of small size DNA having faster mobility compared to native DNA of lane 1. A loss in the fluorescence of DNA was also observed as a function of increase in the concentration of *N*-OH-AABP. This again points towards the damage to the helical structure of DNA.

**Figure 2:**
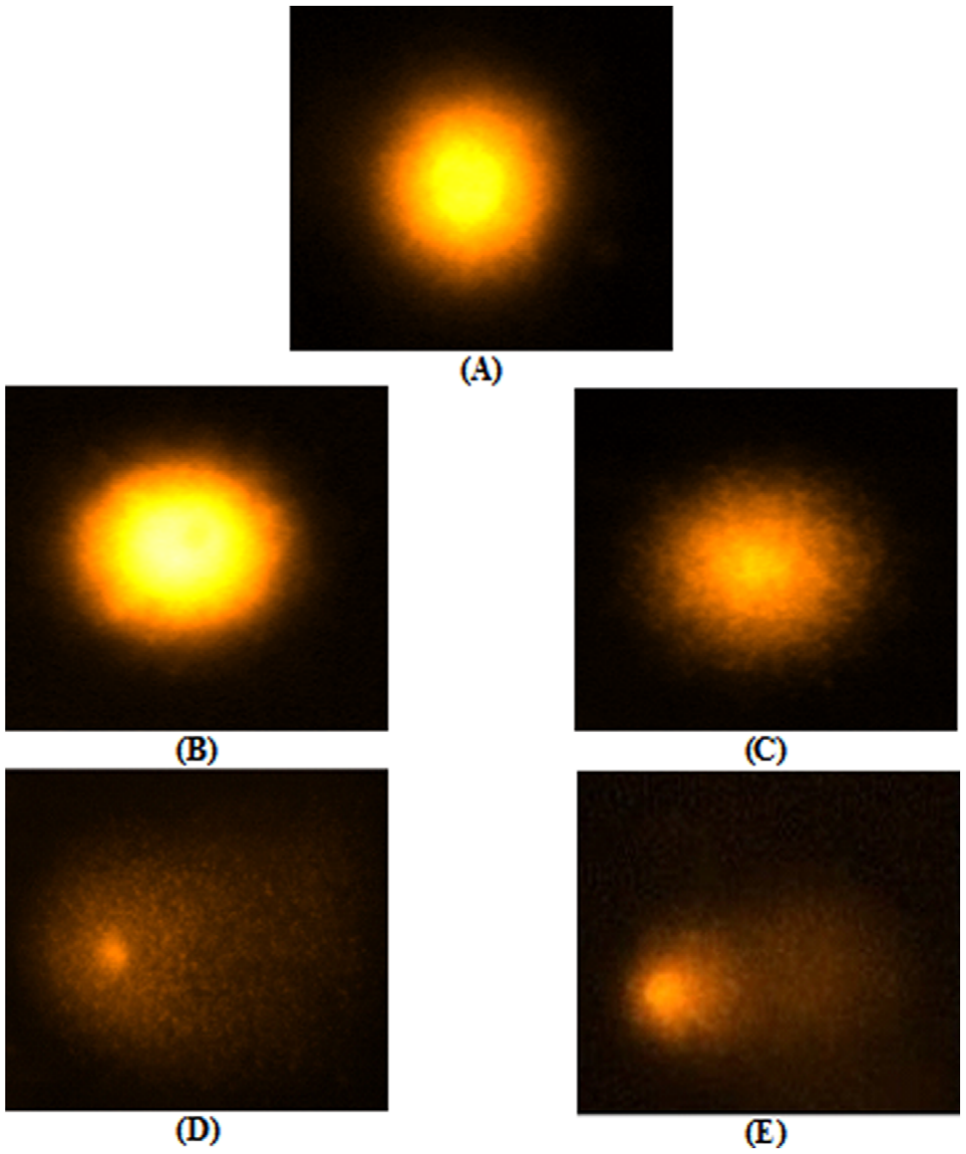
Single cell gel electrophoresis of human lymphocytes showing comets after treatment with the increasing concentrations of *N*-OH-AABP; (A) untreated, (B) treated with 0.378 mM *N*-OH-AABP, (C) with 0.757 mM *N*-OH-AABP, (D) with 1.136 mM *N*-OH-AABP, (E) with 1.515 mM *N*-OH-AABP. All the lymphocytes were treated for 24 hour at 37°C.

The single-cell gel electrophoresis (SCGE) or comet assay is a very sensitive method for evaluating DNA damage. During electrophoresis the damaged DNA migrates from the nucleus towards the anode, forming a shape of a “comet” with a head (cell nucleus with intact DNA) and a tail (relaxed and broken DNA). Therefore, comet assay can be used to test the genotoxicity of carcinogenic agents in cultured cells including freshly isolated human lymphocytes. In this study we have used the comet-assay to analyse the damage to human lymphocyte DNA upon *N*-OH-AABP treatment. The comet pictures, upon treatment of lymphocytes with different concentrations of *N*-OH-AABP, are presented in Figure 2. A comet with a tail is indicative of DNA breakage in single cell gel electrophoresis. Our results clearly established DNA damage upon treatment with 1.515 mM *N*-OH-AABP, as evident from the formation of distinct tail from the diffused head (Figure 2E). We observed that with increasing concentrations of *N*-OH-AABP, the percent DNA in tail also increased. At 0.378 mM concentration of *N*-OH-AABP, 19% of DNA was found in tail. However, at 0.757 mM and 1.136 mM of *N*-OH-AABP the percentage of tail DNA increased to 27% and 58% respectively. It further increased to 89% at 1.515 mM *N*-OH-AABP. The DNA damage parameters, i.e., olive tail moment (OTM) and tail length were also measured and found to be significantly increased with 1.515 mM *N*-OH-AABP as compared to 0.378 mM, 0.757 mM and 1.136 mM *N*-OH-AABP. A marked increase in OTM (94%) was observed in the lymphocytes treated with 1.515 mM *N*-OH-AABP when compared to control (untreated lymphocytes). Furthermore, a substantial increase in tail length was also recorded. It was found to be 72% above that of untreated lymphocyte control. The results are summarized in Table 1.

**Table 1:** Various DNA damage parameters to assess the genotoxic effect of *N*-OH-AABP.

Concentration of *N*-OH-AABP usedto treat lymphocytes (mM)	Percent Tail DNA	Olive tail moment	Tail length (µm)
0	7.9	0.25	14.6
0.378	11	0.33	18.1
0.757	22.8	0.9	22.8
1.136	56.8	1.09	31
1.515	89.2	3.8	55.8

In an earlier publication, we observed 62% hyperchromicity at 260 nm in the UV absorption spectrum of *N*-OH-AABP modified human DNA [32]. The observed hyperchromicity represents exposure of chromophoric groups as a result of generation of strand breaks and dissociation of hydrogen bonds in the case of modified DNA.

**Figure 3:**
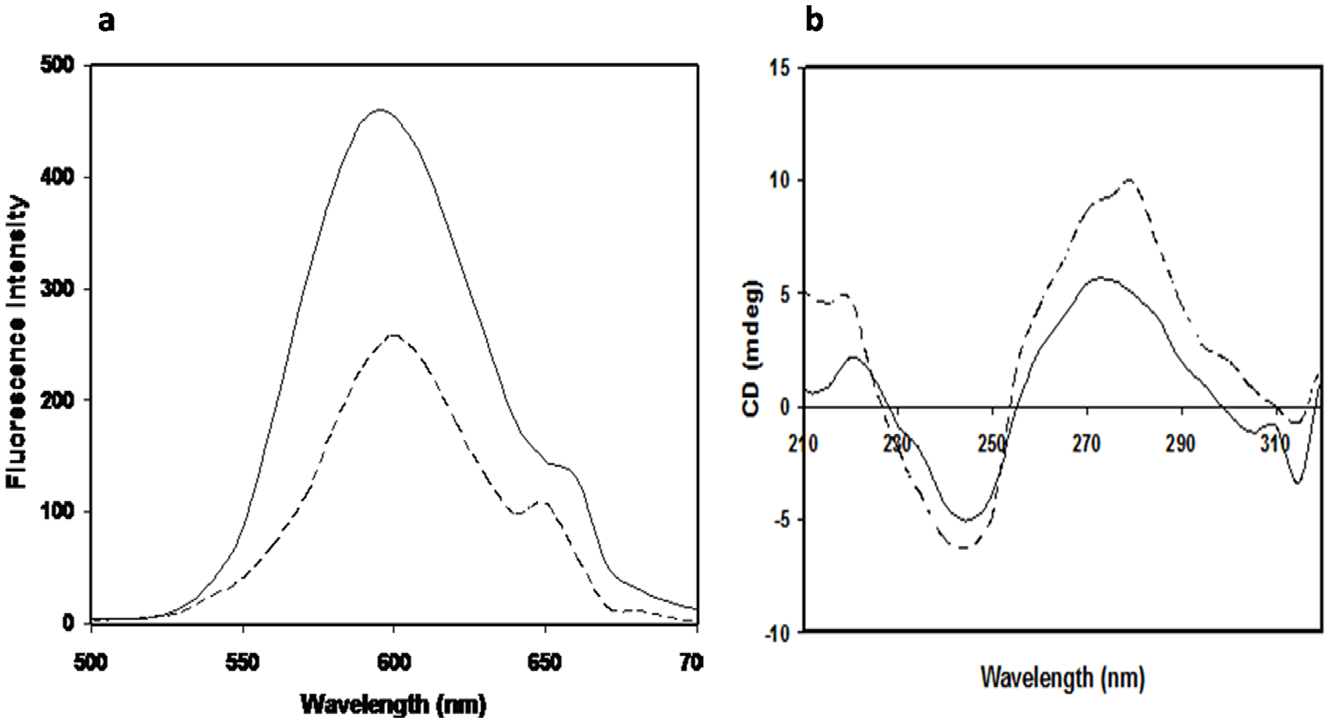
Fluorescence emission spectra of native human DNA (–––) and modified human DNA with 1.515 mM *N*-OH-AABP (–) **[a]**. Circular dichroic spectra of native human DNA (–) and *N*-OH-AABP modified human DNA (––––) **[b]**.

Neither native DNA nor its modified form has its own fluorescence and therefore an extrinsic fluorophore, ethidium bromide, was used for fluorescence spectroscopy of DNA and its *N*-OH-AABP-modified form. Native and *N*-OH-AABP modified DNA were incubated with ethidium bromide for 30 min and the emission profile was recorded using excitation wavelength (325 nm) of ethidium bromide (Figure 3A). A decrease of 43.17% in the fluorescence intensity of modified DNA, as compared to its native form, signifies perturbations in DNA double helical structure as a result of *N*-OH-AABP modification.

Changes in the DNA structure were evaluated by ellipticity measurements. The *N*-OH-AABP modified DNA exhibited a 5 nm shift (from 275 to 280 nm) in the CD signal along with an increase in ellipticity from 5.57 to 9.27 mdeg (Figure 3B), indicating structural changes in the DNA molecule. The increase in ellipticity corresponds to 39.9% loss in the DNA structure upon modification. This structural loss may be due to unstacking of DNA bases as a result of helix destabilization. The structural perturbations suggest unfolding of DNA, may be due to the generation of single strand breaks.

**Figure 4:**
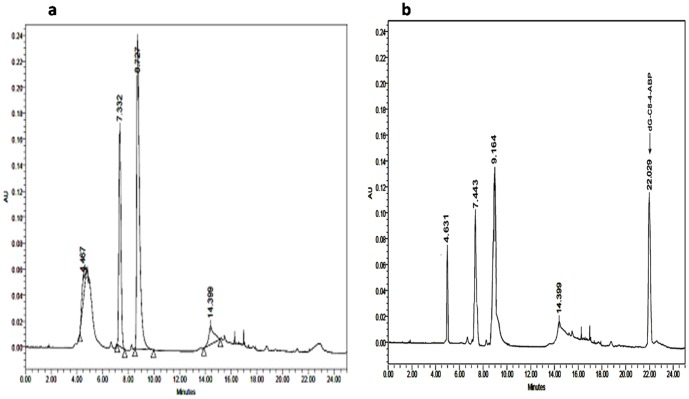
Representative HPLC chromatogram of acid hydrolysate of native human DNA [a]. Representative HPLC chromatogram of acid hydrolysate of *N*-OH-AABP modified human DNA **[b]**.

Figure 4A and 4B show representative HPLC chromatograms of acid hydrolysed samples of native and *N*-OH-AABP modified human DNA respectively. Well defined peaks at retention time 4.467 min, 7.332 min and 8.727 min were observed in native human DNA. However, in the case of modified DNA these peaks shifted to 4.631 min, 7.443 min and 9.164 min respectively, suggesting modification of the DNA bases. The extra peak at a retention time of 22.029 min in the acid hydrolysate of modified DNA is characteristic of dG-C8-4-ABP adduct, a known marker for DNA damaged by 4-ABP and *N*-OH-AABP [33]. The identification of dG-C8-4-ABP adduct is in agreement with the published reports on DNA-adducts with arylamines in experimental animals [15]–[17]. Similar DNA-adducts have been reported in the biopsy samples of human urinary bladder [18], [19].

Female rabbits immunized with *N*-OH-AABP modified human DNA showed vigorous humoral response eliciting high titre immunogen specific antibodies. The experimentally induced antibodies were used as an immunochemical probe to detect the *N*-OH-AABP or related arylamines induced lesions in the genomic DNA of bladder cancer patients. The binding pattern of the DNA isolated from bladder cancer patients was quite revealing in competitive inhibition assay. Inhibition of anti-*N*-OH-AABP-DNA IgG by lymphocyte DNA from bladder cancer patients was recorded in the range of 60.5% to 77.6%. While, the inhibition caused by lymphocyte DNA from normal healthy individuals was quite low (Table 2). Significantly high recognition of the lymphocyte DNA from bladder cancer patients by the experimentally induced antibodies against *N*-OH-AABP modified DNA is a clear indicator of epitope sharing between the genomic DNA of bladder cancer patients and the human DNA modified *in vitro* by *N*-OH-AABP. This leads to the conclusion that *N*-OH-AABP, or a related arylamine, generates neo-epitopes on the DNA molecule that are recognized as ‘*alien*’ or *non-self* by the immune system resulting in autoantibody generation in bladder cancer patients. Significantly high level of recognition of the genomic DNA from bladder cancer patients by the experimentally induced antibodies against *N*-OH-AABP modified human DNA is an evidence towards the involvement of modified bases and single strand regions in the disease pathogenesis.

**Table 2:** Detection of *N*-OH-AABP mediated lesions in the DNA of bladder cancer patients and normal healthy individuals.

Sera group	Number of samples tested	Maximum percent inhibition at 20 µg/ml	Mean ± SD
Bladder cancer*	12	70.2, 60.5, 61.7, 69.6, 65.4, 68.7, 76.8, 69.3, 64.4, 77.6, 73.4, 68.3	68.83±5.4%
Normal human	8	30.4, 25.6, 24.25, 38.4, 34.4, 28.3, 20.85, 29.6	29.0±5.6%

Binding of anti-*N*-OH-AABP modified DNA IgG to DNA isolated from lymphocytes of bladder cancer patients normal healthy individual was ascertained by competitive inhibition ELISA. The microtitre plates were coated with *N*-OH-AABP modified human DNA (2.5 µg/ml).

Data are expressed as means ± SD. Statistical significance was evaluated by 1-way ANOVA. Results were considered significant at P<0.05.
